# Function focused care in hospital: A mixed-method feasibility study

**DOI:** 10.1016/j.ijnsa.2021.100045

**Published:** 2021-09-29

**Authors:** Selma Kok, Janneke M. de Man-van Ginkel, Carolien Verstraten, Barbara Resnick, Silke F. Metzelthin, Nienke Bleijenberg, Lisette Schoonhoven

**Affiliations:** a Julius Center for Health Sciences and Primary Care, University Medical Center Utrecht, Utrecht University, Heidelberglaan 100, Utrecht 3584 CX, the Netherlands; b University of Applied Sciences Utrecht, Heidelberglaan 7, Utrecht 3584 CS, the Netherlands; c University of Maryland, 655W. Lombard St., Baltimore, MD 21201, United States; d Department of Health Services Research, Care and Public Health Research Institute (CAPHRI), Faculty of Health, Medicine and Life Sciences, Maastricht University, Postbus 616, Maastricht 6200 MD, the Netherlands; e School of Health Sciences, Faculty of Environmental and Life Sciences, University of Southampton, University Road, Southampton, SO17 1BJ, United Kingdom

**Keywords:** Activities of Daily Living [MeSH], Feasibility studies [MeSH], Function Focused Care, Functional decline, Hospitals [MeSH], Nursing staff, Hospital [MeSH], Mobility, Nursing [MeSH], Nursing care [MeSH], Patient-centered care [MeSH]

## Abstract

**Background:**

During hospitalization patients frequently have a low level of physical activity, which is an important risk factor for functional decline. Function Focused Care (FFC) is an evidence-based intervention developed in the United States to prevent functional decline in older patients. Within FFC, nurses help older patients optimally participate in functional and physical activity during all care interactions. FFC was adapted to the Dutch Hospital setting, which led to Function Focused Care in Hospital (FFCiH). FFCiH consists of four components: (1) ‘Environmental and policy assessment’; (2) ‘Education’; (3) ‘Goal setting with the patient’ and (4) ‘Ongoing motivation and mentoring’. The feasibility of FFCiH in the Dutch hospital setting needs to be assessed.

**Objective:**

Introduce FFCiH into Dutch hospital wards, to assess the feasibility of FFCiH in terms of description of the intervention, implementation, mechanisms of impact, and context.

**Design:**

Mixed method design

**Setting(s):**

A Neurological and a Geriatric ward in a Dutch Hospital.

**Participants:**

56 Nurses and nursing students working on these wards.

**Methods:**

The implementation process was described and the delivery was studied in terms of dose, fidelity, adaptions, and reach. The mechanisms of impact were studied by the perceived facilitators and barriers to the intervention. Qualitative data were collected via focus group interviews, observations, and field notes. Quantitative data were collected via evaluation forms and attendance/participation lists.

**Results:**

A detailed description of FFCiH in terms of what, how, when, and by whom was given. 54 Nurses (96.4%) on both wards attended at least 1 session of the education or participated in bedside teaching. The nurses assessed the content of the education sessions with a mean of 7.5 (SD 0.78) on a 0–10 scale. The patient files showed that different short and long-term goals were set. Several facilitators and barriers were identified, which led to additions to the intervention. An important facilitator was that nurses experienced FFCiH as an approach that fits with the principles underpinning their current working philosophy. The experienced barriers mainly concern the implementation elements of the FFCiH-components ‘Education’ and ‘Ongoing motivation and mentoring’. Optimizing the team involvement, improving nursing leadership during the implementation, and enhancing the involvement of patients and their family were activities added to FFCiH to improve future implementation.

**Conclusions:**

FFCiH is feasible for the Dutch hospital setting. Strong emphasis on team involvement, nursing leadership, and the involvement of patients and their families is recommended to optimize future implementation of FFCiH in Dutch hospitals.

**What is already known**

• Function Focused Care is an evidence-based intervention that prevents functional decline among hospitalized elderly;
• It is proven to be effective in assistant living facilities, nursing homes, home care, and acute care settings in the United States;
• It is unknown if the intervention can be implemented in the Dutch Hospital Setting.

**What this paper adds**

• This study showed the feasibility of Function Focused Care in Hospital and the challenges in some elements of the intervention;
• Optimizing the team involvement, improving nursing leadership during the implementation, and enhancing the involvement of patients and their family were added to FFCiH to improve future implementation.
• This feasibility study can guide the use of process evaluation in examining the feasibility of an intervention in daily practice.

## 1 Background

The philosophy in nursing care focuses on helping patients obtain and maintain their overall health, functional status, and physical activity (Boltz et al., 2014); Englebright et al., 2014; Kirkevold, 1997, 2010; Kitson et al., 2013a). In their daily care, nurses assist patients with their Activities of Daily Living (ADLs) and mobility, which is one of the essential nursing care activities (Kitson et al., 2010; Zwakhalen et al., 2018) In hospital, patients often experience functional decline caused by diseases such as stroke (Dhamoon et al., 2012). Furthermore, a hospital admission itself is burdensome especially for geriatric patients, and is associated with poor health outcomes after admission (Buurman et al., 2011). During hospitalization patients frequently have a low level of physical activity (Brown et al., 2009; Fini et al., 2017; Floegel et al., 2018; Pedersen et al., 2013; Resnick et al., 2015), which is an important risk factor for functional decline (Hoogerduijn et al., 2014; Zisberg et al., 2015). There are diverse challenges that limit physical activity, including patient factors, environmental and policy issues, and medical and nursing interventions (Brown et al., 2007; Resnick and Boltz, 2019).

Function Focused Care (FFC) was developed to overcome the challenges of engaging older patients in physical activity when hospitalized. FFC is a philosophy of care in which nurses work with patients to optimize function and physical activity during all care interactions (Resnick et al., 2012). Previous research provides evidence that FFC is safe and has positive effects on functional status, physical activity, mobility, and ADLs in patients living in the community or nursing homes (Galik et al., 2013; Resnick et al., 2021, 2013; Verstraten et al., 2020). Also, studies in acute care have shown promising results regarding functional status and mobility in older hospitalized patients (Boltz et al., 2015, 2012, 2011; Resnick et al., 2016). Examples of nursing care according to the FFC approach include having nurses walk patients to the bathroom, or to taking meals while sitting at the table rather than remaining in bed (Galik et al., 2013; Resnick et al., 2012, 2013, 2011). The essence of FFC is to optimize and maintain function and increase patients’ time spent in physical activity (Galik et al., 2013; Resnick et al., 2012, 2013, 2011, 2009). During the entire hospital admission period, the patient is encouraged to be actively engaged in all daily care activities at a level tailored to the patient (Galik et al., 2013; Resnick et al., 2012, 2013, 2011). FFC is an interesting approach to implement and evaluate in the Dutch hospital setting. However, it is not possible to simply generalize previous findings to the Dutch hospital setting, because of the major differences in health care systems in the U.S. and the Netherlands. For example, mobilization policies are different (Bakker et al., 2014; Boltz et al., 2010; Buurman, 2015; Lorgunpai et al., 2020) and staffing ratios of patients to nurses are lower in American than in Dutch hospitals (Aiken et al., 2012).

With the guidance of the development phase of the Medical Research Council (MRC) guidance for developing and evaluating complex interventions (Craig et al., 2008) a working group, consisted of researchers and hospital care nurses, expert opinion was obtained of the necessity of adapting FFC for their daily practice. The working group concluded that no adaptions were needed in the content of FFC. When implementing FFC in Dutch hospitals, the variation in daily practice in nursing care and multidisciplinary collaboration between hospitals or wards, such as mobilization policies, electronic medical/nursing records, and policy regarding nursing reports, need to be taken into account. Therefore, the working group developed a guideline and an educational program for implementing and providing FFC in the Dutch hospital care setting, referred to as Function Focused Care in Hospital (FFCiH).

To ensure that FFCiH, including the guideline and educational program, optimally fits the daily practice of the Dutch hospital care, an evaluation of the feasibility is recommended in the MRC-framework as a next step (Craig et al., 2008). There is no widely used definition of feasibility, but in general, it is used to gain insight into the feasibility of study procedures, methodology used in trials, and the intervention to be evaluated (Arain et al., 2010). In our study, we examine the feasibility of the intervention when applied in the daily care of the Dutch hospital setting. To understand the feasibility a process evaluation can have a vital role (Moore et al., 2015). Therefore we conducted a process evaluation using the guidance of process evaluation of complex interventions (Moore et al., 2014, 2015). According to Moore's guidance (2014), the key functions of process evaluation are (1) Description of the intervention and its causal assumptions; (2) the process through which interventions are delivered, and what is delivered in practice (implementation); (3) the intermediate mechanism through which intervention activities produce effects (mechanism of impact); (4) factors external to the intervention which may influence its implementation, or whether it mechanisms of impact act as intended (context), see Table 1. Therefore, the aim of this study is to introduce FFCiH into Dutch hospital wards and to assess the feasibility of FFCiH in daily hospital care.

**Table 1:** Feasibility of Function Focused Care in Hospital (FFCiH)

Key elements†		Definition†	Operationalisation		Data sources													
					FG		MM		Ob		PF		AL		EF		FN	
**Description of the intervention and its causal assumptions**			The intervention is described using the Template for Intervention Description and Replication (TIDieR)															
**Implementation**		The process through which interventions are delivered, and what is delivered in practice																
Implementation process		The structures, resources and mechanisms through which delivery is achieved																
Delivery		How is delivery achieved, and what is actually delivered																
	Dose	The quantity of delivery		Whether all four components of FFCiH were actually providing.		x		x		x		x						
	Fidelity	The quality of delivery		Satisfaction with the initial training (component: education)												x		
				The perspective of the nurses to all components of FFCiH		x		x		x								
	Adaptions	Adaptions made during implementation		Identified and reported during implementation														x
	Reach	The extent to which a target audience comes into contact with the intervention		The percentage of the nursing staff that attended to the initial training. (component: education)										x				
				The percentage of goals in the patients’ electronic files. (component: goal setting with the patient)								x						
				The percentage of the nursing staff that received bedside teaching. (component: ongoing motivation and mentoring)						x								
**Mechanism of impact**		The intermediate mechanism through which intervention activities produce intended (or unintended) effects																
Participants responses to and interactions with the intervention		How participants interact with a complex intervention		Perceived facilitators and barriers regarding each of the four FFCiH components.		x		x		x								
				Satisfaction with the initial training (component: education)												x		
**Context**		Factors external to the intervention which may influence its implementation, or whether it mechanisms of impact act as intended		Facilitators and barriers external to FFCiH that will influence the implementation FFCiH				x		x						x		

Abbreviations: FG, focus group interviews; MM, monthly meetings with FFCiH coaches; Ob, observations or comments from nurses/patients during bedside teaching; PF, monitoring patients’ files; AL, attendance list; EF, evaluation form; FN, field notes.

† key elements guided by the MRC framework for process evaluation (Moore et al., 2015)

## 2 Method

### 2.1 Design

To obtain an in-depth understanding of the feasibility, we used a convergent parallel mixed-methods design, in which qualitative and quantitative data were collected simultaneously. This study was conducted from October 2015 until February 2016 by a researcher with a background in nursing, who also fulfilled the role of the implementer. The study is presented using the Standards for Quality Improvement Reporting Excellence (SQUIRE 2.0) checklist (Ogrinc et al., 2015).

### 2.2 Population and sample

The study was conducted on a neurological and geriatric ward of a general hospital in the middle of the Netherlands. Each ward participated for three months. To be included in the study the nurse needed to be working at these wards caring for stroke and geriatric patients. No exclusion criteria were used for receiving bedside teaching and for attending the focus group interview. Convenience sampling was used for bedside teaching and focus group interviews.

### 2.3 Function focused care in hospital (FFCiH)

**Fig. 1:**
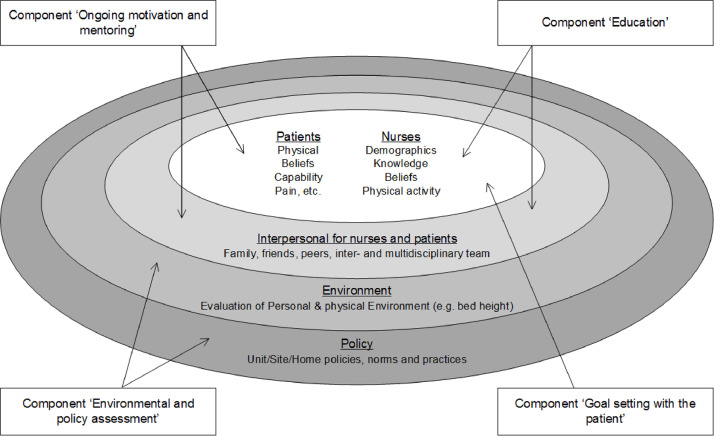
Function Focused Care in Hospital (based on Resnick et al. (2012). Restorative Care Nursing for Older Adults. A Guide for All Care Settings. Springer Publishing Company, New York.).

Within FFCiH, implementation and intervention elements are closely integrated and cannot be viewed separately from each other (Resnick et al., 2012). Primarily, FFCiH concerns (1) the patients and nurses; (2) the patients’ family and the multidisciplinary team; (3) the wards’ environment, and (4) the policy and culture of the ward. Furthermore, FFCiH consists of four important components, the key components: (a) ‘Environmental and policy assessment’; (b) ‘Education’; (c) ‘Goal setting with the patient’; (d) ‘Ongoing motivation and mentoring’. The component ‘Environmental and policy assessment’, which includes suitability of the policy, culture, and environment, is mainly directed to the wards’ environment and the policy and culture of the ward. In the component ‘Education’ the nurses are educated and they have trained the patient and informed the family and the multidisciplinary team. This component mainly affects patients and nurses and the patients’ family and the multidisciplinary team. The component ‘Goal setting with the patient’ is directed to the patients and nurses: patients and nurses set goals together, based on the physical, psychological possibilities, and preferences of the patient. The component 'Ongoing motivation and mentoring' concerns the nurses who, in close collaboration with the multidisciplinary team, are motivated and have learned how to motivate the patients and their families and increase the patients’ self-confidence. This component is therefore directed to the patients and nurses and the patients’ family and the multidisciplinary team (Fig. 1).

The intervention is described using the Template for Intervention Description and Replication (TIDieR) to improve the completeness of reporting and replicability of FFCiH (Hoffmann et al., 2014). All aspects of FFCiH are described in terms of what, why, how, when, who, and how much in Table 2.

**Table 2:** Description of the components and additions* of Function Focused Care in Hospital setting (FFCiH).

		*Description items*†				
*Components FFCiH^‡^*		What	Why	How	When, Who and how much	Modifications made during study
Environmental and policy assessment	implementation	Discuss if the (ward) policy and environment are supportive to physical activity of patients (e.g. general policy regarding what in the team the general objective is for the nursing care, the availability of mobility devices, lunch meetings). Decide where goals and their evaluation need to be noted in the patients’ files. Record this information in the training and FFCiH-guideline.^§^	To tailor FFCiH to the ward/ make adaptations if necessary for a successful implementation. To create a safe and attractive environment that supports the patients to be physically active.	By observations and discussions.	Before the initial training by the ward manager, FFCiH coach and researcher. The researcher initiates the meetings. The ward manager or coaches take care of the adaptations to the patient files and communication with the team.	
	intervention	Remove obstacles. Stimulate patients to wear clothing and shoes during the day. Stimulate patients to use the necessary devices.	To ensure a safe environment that supports the patients to be physically active.	By observation. Face-to-face with patient.	During all daily nursing care activities. Provided by nursing staff.	
Education	implementation	Preparation: Emphasize involvement of ward manager. Set a percentage of nurses that need to be trained. Identify and appoint FFCiH coaches and inform them about their role. Select coaches who are registered nurse with experience in care. Discuss when and how to inform (multidisciplinary) team about FFCiH. Training: Consists of theory of FFCiH; goal setting with the patient; motivation techniques to encourage the patient to be more psychically active; role-playing to practice above items; questions / discussion. Emphasize patient and familyinvolvement. Follow up training. Emphasize patient and family involvement.	Preparation: To train a predefined number of the nursing staff. To have FFCiH coaches to cover different shifts, prepared for their role. To tailor bedside teaching to the needs of the nursing staff. To inform the multidisciplinary team in time. Training: To train and motivate the nursing team to apply FFCiH. Follow up training: To refresh training and discuss (time-related) barriers.	Preparation: Via email and/or multidisciplinary meetings and face-to-face. Training: During 4 training sessions of half an hour; invite other disciplines (physiotherapist, occupational therapist).	Preparation: Before the training by the ward manager, FFCiH coaches and researcher. Training: In the first week(s) of the implementation phase. The researcher provided the training. Information is provided to those who do not attend the training by one-on-one sessions or guideline^§^. Follow up training if necessary, provided by researcher or FFCiH coaches.	The four sessions were combined to 1 or 2 sessions with a total of 2 hours depending on the possibilities of the ward. The manager scheduled the nurses (max 15/group) to the training and motivated them to attend the training. Training sessions outside the wards.
	intervention	Provide Information about FFCiH to patients and family	To enhance patients' motivation for active engagement in their ADLs	Face-to-face with patients and/or family.	At admission and during all daily nursing care activities. Provided by nursing staff.	
Goal setting with the patient	implementation	Inform the multidisciplinary team about the patients’ goals (where to find them in the files).	To enhance communication about the patients’ goals within the team.	Face-to-face, by email and during multidisciplinary meeting.	At the beginning of the implementation. By the ward manager and/or FFCiH coaches.	
	intervention	Set one long-term goal and daily short-term goals in collaboration with patients (or family), except those who are in terminal care. Goals are focused on physical activity/ADLs and based on the evaluation of patients’ ability in physical activity of all disciplines, what is important to the patients and her/his energy balance. Establish an individualized plan.	To enhance patients' motivation for active engagement in their ADLs. To increase tailored individual patient-centred care.	Face-to-face with patients and/or family.	Within 24 hours of admission. Daily goals are evaluated at a daily bases and reported in the patient's file and updated as indicated. Provided by nursing staff. Family will be asked to support the patient achieving daily goal.	
Ongoing motivation and mentoring	implementation	Staff motivation: State how much time will be needed for bedside teaching and the best time to do this and by whom. Inform nurses and other disciplines about the benefits for patients of physical activity and goal setting in close collaboration with the patient**.** Give verbal encouragement and compliments. Discuss facilitators and barriers associated with FFCiH. Help nurses to apply FFCiH into their daily practice with emphasis on patient and family involvement. Motivation of FFCiH coaches: In establishing an implementation plan for sustainability. In applying their role (motivating of colleagues, being a role model, observing potential barriers)	Staff motivation: To motivate nursing team to apply FFCiH. To identify and reduce barriers for applying FFCiH. To mentor the FFCiH coaches to get familiar with their role.	Staff motivation: Bedside teaching (face-to-face), follow up training sessions and by monthly emails Mentoring of the FFCiH coaches face-to-face and during monthly meetings	Staff motivation: Bedside teaching once or twice a week by the researcher/FFCiH coaches. FFCiH coaches send monthly emails to the team. Ward manager provides (time) conditions and supports the nursing team and coaches. Monthly meetings with FFCiH coaches, ward manager and researcher.	
	intervention	Patient motivation: Inform patients and family about the risks of inactivity and advantages of activity. Give verbal encouragement. Identify and reduce unpleasant experiences. Give cues that can trigger movements.	To enhance patients’ motivation for active engagement in their ADLs and to increase time spent in physical activity.	Face-to-face with patients and/or family. Written information to inform patients and family.	At admission and during all daily nursing care activities. Provided by nursing staff. Encouragement can also be done by friend or family member (social support).	

*Additions made based on the feasibility study are in underlined font

† Description items using the TIDieR framework (Hoffmann et al., 2014) ^‡^All four FFCiH components comprise intervention as implementation elements. ^§^All components are described in a guideline (in Dutch; available upon request) to support the manager, FFCiH coaches and nursing staff during the implementation phase and to apply FFCiH.

### 2.4 Measures

Descriptive data from the wards' nurses were collected such as age, sex, registered nurse or nursing student, and years working at the ward.

#### 2.4.1 Feasibility outcomes

We studied four key elements of process evaluation to assess the feasibility: the description of the intervention, the implementation, the mechanism of impact, and the context (Moore et al., 2015). With the definition of Moore et al. (2014) we operationalized these elements, see Table 1.

The implementation was described by the implementation process and delivery, which was defined in terms of dose, fidelity, adaptions, and reach (Moore et al., 2015). The implementation process presents the procedures and resources through which delivery is achieved. We defined dose as actually delivering the four components, which was observed and noted in the logbook by the researcher. Reach was measured by the percentage of the nursing staff that attended the education sessions (component ‘Education’), the percentage of the nursing staff that received bedside teaching (component ‘Ongoing motivation and mentoring’), and the goals set in the electronic patients’ files (component ‘Goal setting with the patient’). Fidelity was defined as the quality of what was delivered and measured by an evaluation form of the education sessions (component ‘education’) and the perspective of the nurses to all components of FFCiH. Adaptions made during implementation were reported into the logbook.

The mechanisms of impact were defined as how participants interact with a complex intervention (Moore et al., 2015). We identified the interactions with the intervention by the perceived facilitators and barriers of the nurses regarding each of the four FFCiH components and the satisfaction with the education sessions (component ‘Education’).

The contextual factors were defined as facilitators and barriers external to FFCiH that will influence the implementation of FFCiH. Nurses, FFCiH coaches, and ward managers were asked about these factors, which were noted in the logbook.

### 2.5 Procedures of data collection

In the first two weeks, the education sessions were planned with the ward manager and FFCiH coaches. During the education sessions (component ‘Education’) the attendance was listed. After the education sessions, ward nurses were asked to fill in an evaluation form to rate the content and teaching method of the education sessions and the trainer on a scale of 0 (very bad) to 10 (excellent).

Participation in bedside teaching of the nurses (component ‘Ongoing motivation and mentoring’) was noted next to the attendance. Bedside teaching started after the completion of the initial education sessions. If and how often patients’ goals were reported was checked after three months. Furthermore, we monitored the nursing reports to assess if and how patients were motivated for physical activity and if goals were evaluated and adapted.

Field notes were made by the researcher (CV) in a logbook of (1) observations and experiences of the care provided by the nurses during bedside teaching, (2) monthly meetings with the FFCiH coaches and (3) specifics about nursing reports and patients goals. Field notes of the bedside teaching were made immediately after working with the nurse and were checked by that nurse.

Focus group interviews were held at the end of the three-month implementation process. Nurses were invited to attend the focus group via email. The focus group interviews lasted 1 h and were audio-taped on the neurological ward. The focus group on the geriatric ward was not audiotaped, since it has been conducted ad hoc during a regular team meeting. A semi-structured interview guide with open questions, based on what was seen in the questionnaires and observations, was used (Table 3). In Table 1, an overview is given of which data source was used for collecting the data for each of the outcomes.

**Table 3:** Topic list for focus group interview*

1	What are the reasons and/or barriers to not set goals with the patient or their family?
2	The question “fellow nurses don't apply FFCiH” is mentioned as barrier. What is the influence of fellow nurses?
3	The question “I have read or remembered the information about FFCiH (handbook, sheets, training) not thoroughly enough” is not fulfilled very variable. Who has an explanation for this?
4	What did you think of the implementation (training, bedside teaching, meetings coaches, regular mails): tips and tops?
5	What actions to motivate did you use? (explain, patient cards, demonstrate, ask family, compliment, reduce pain etc.)
6	How do you inform patients and their family?
7	What do you think about the comment ‘I'm afraid to ask if they walk with me, they're so busy’?
8	What is your overall opinion about FFCiH?

*based on observations and questionnaires

### 2.6 Analyses

#### 2.6.1 Quantitative analysis

With the quantitative data, descriptive analyses were conducted using IBM SPSS 25. Age and satisfaction with the education sessions were calculated as mean, standard deviation, and range. The numbers and percentages of nurses’ sex and education, and (registered) nurses who participated in bedside teaching, were motivated, helped with goal setting, received extra information, and participated in education sessions (min. 1 session), bedside teaching, or completed a questionnaire were calculated. After three months, the number of goals in the patient files was counted.

#### 2.6.2 Qualitative analysis

The qualitative data were analyzed using Excel following the method of thematic analysis (Braun and Clarke, 2006), conducting four steps. (1) During the first step focus groups were transcribed by the researcher (CV). Field notes were checked for completeness by one of the participants of the focus group. The logbook made of the bedside teaching was used as a transcript of observations of the daily care. (2) The transcripts were coded per data resource by two researchers individually (CV & SK). Consensus about codes was reached by consulting a third researcher (JMG). (3) Data syntheses started by clustering the codes into subthemes and were all discussed by two researchers (SK, JMG), to provide an overall view of the different findings. (4) The subthemes were clustered according to the key elements of process evaluation: implementation process, mechanism of impact, and context. Data triangulation was used to assess the completeness and accuracy of data.

#### 2.6.3 Data synthesis

As a final step, we merged these findings. This resulted in additions needed to optimize the FFCiH for the Dutch hospital setting.

### 2.7 Ethical approval

The study was approved by the Medical Research Ethics Committee of the University Medical centre Utrecht (approval number 15/517). The ward managers consented to participate after they had received verbal and written information regarding the intervention and study. All nurses working at one of these wards were approached during regular team meetings and via email and gave informed consent for participation in the different study activities: education sessions, bedside teaching and focus group interviews.

## 3 Results

All registered nurses (RNs) and nurse students working in the neurological ward and the geriatric ward (*n* = 56) gave informed consent. A total of 54 (96.4%) nurses participated in the study by attending the education sessions or receiving bedside teaching. The mean age was 32.3 (SD 11.1, range 19.0–55.0). The mean years working at the ward was 7.3 (SD 5.9, range 0.5–23.0). From these nurses, eight RNs from the neurological ward, and three RNs, and two nurse students from the geriatric ward attended the focus group interviews (Table 4).

**Table 4:** Baseline characteristics, reach and fidelity outcomes

	**Total sample**		**Ward**			

**Characteristics**	Inclusion, *n* = 56		GeriatricInclusion, *n* = 17(31.5%)		NeurologicalInclusion, *n =* 39(68.5%)	
**Baseline characteristics**						
female sex *n (%)*	50	(89.3)	14	(82.4)	36	(92.3)
Age *m* (SD) (range)	32.3	(11.1) (19.0-55.0) (n=36^a^)	29.8	(12.0) (19.0-55.0) (n=13^a^)	33.7	(10.6) (19.0-55.0) (n=23^a^)
Years working at ward *m* (SD) (range)	7.3	(5.9) (0.5-23.0) (n=29^a^)	5.6	(4.9) (1.0-14.0) (n=8^a^)	8.0	(6.2) (0.5-23.0) (n=21^a^)
**Reach and fidelity outcomes**						
Registered nurses *n* (%)	44	(78.6)	10	(58.8)	34	(87.2)
Registered nurses that followed education *n* (%)	33	(75.0) (n=44)	8	(80.0) (n=10)	25	(73.5) (n=34)
Registered nurses that participated in bedside teaching, were motivated, helped with goal setting or received extra information *n* (%)	20	(45.5) (n=44)	5	(50) (n=10)	15	(46.9) (n=32)
Nursing students *n* (%)	12	(20.3)	7	(42.2)	5	(13.5)
Nursing students that followed education *n* (%)	7	(58.3) (n=12)	6	(85.7) (n=7)	1	(20.0)
Nursing students that participated in bedside teaching, were motivated, helped with goal setting or received extra information *n* (%)	7	(58.3) (n=12)	7	(100) (n=7)	0	(0)
Total of nurses and nursing students that followed education *n* (%)	40	(71.4)	14	(82.4)	26	(66.7)
Total of nurses and nursing students that participated in bedside teaching *n* (%)	27	(48.2)	12	(70.6)	15	(38.5)
Total of nurses and nursing students that participated in education (min 1 session) or bedside teaching *n* (%)	54	(96.4)	17	(100)	37	(94.9)
Satisfaction education						
Content m (SD)	7.5	(0.78)				
Working method m (SD)	7.4	(0.69)				
Teacher m (SD)	8.0	(0.65)				

a not collected for all included nurses

### 3.1 Implementation

#### 3.1.1 Implementation process

The intervention was introduced to both wards individually by the researcher. For the component ‘Environmental and policy assessment’ both teams decided the team managers be the best person to complete the environmental and policy assessment. In both wards, this assessment showed that no changes were needed in the policies. For the component ‘Education’, five FFCiH coaches for the geriatric ward and six for the neurological ward were appointed in the first week, to cover different shifts during the week. The FFCiH coaches were nurses from the wards who supported their colleagues to incorporate FFCiH into routine care. For the component ‘Environmental and policy assessment’ the FFCiH coaches determined on which place in the electronic patient file the goals and their evaluation should be noted. They communicated this to their colleagues by email and added the information to their FFCiH guideline.

In the first two weeks, the component ‘Education’ continued by informing and educating the teams by the researcher. Beforehand, the ward managers from both wards set goals for the percentage of nurses needed to be trained: 60% (*n* = 39) of the neurological nursing staff and 100% (*n* = 17) of the geriatric nursing staff. Finally, in the first week, the ward managers sent an email to inform the multidisciplinary team about the implementation of FFCiH on their ward and they were invited as well to the education sessions including an occupational therapist (neurological ward), two activity leaders (geriatric ward), and four physiotherapists (two from each ward). After the education sessions, the FFCiH guideline was provided for both wards individually, nurses started with assessing the environment of patient rooms (component ‘Environmental and policy assessment’), informing the multidisciplinary team (component ‘Education’) and goal setting with patients (component ‘Goal setting with the patient’). Follow-up education sessions were provided by the researcher as needed to refresh the information from the education sessions.

For the component ‘Ongoing motivation and mentoring’, after the education sessions, the researcher provided bedside teaching once or twice a week at each of the wards during the entire three months, approaching the nurses who were working at the ward that day. Second, during monthly meetings and one-on-one meetings, the FFCiH coaches were trained and mentored by the researcher and the implementation process was discussed. Third, the FFCiH coaches motivated and supported their colleagues to perform FFCiH.

#### 3.1.2 Delivery

Regarding the component ‘Environmental and policy assessment’ most of the items were delivered as intended. The ward manager observed awareness of FFCiH in the team, as team members often discussed the application of FFCiH in their regular communications during the day. The role of the ward manager was not clear to the nurses and communication between the FFCiH coaches and ward manager could be better according to the nurses. Furthermore, the FFCiH coaches suggested in a monthly meeting that they did not get enough time for the implementation of FFCiH. One nursing student stopped being a coach, because of the lack of time she got. One of the ward managers was unwilling to make the ward more attractive for mobilization of patients, for example by providing hints and clues on the walls of the wards’ hallway.

Overall, the component ‘Education’ was delivered as intended according to the nurses. The nurses assessed the content of the education sessions with a mean of 7.5 (SD 0.78), the working method with a 7.4 (SD 0.69), and the teacher with an 8.0 (SD 0.65) (Table 5). Also, when a nurse did not follow the initial education sessions, they were instructed by the FFCiH coaches or the researcher. Adaptions made during the implementation were only related to the initial education sessions. The four sessions were combined into 1 or 2 sessions with a total of 2 h depending on the possibilities of the ward. The manager scheduled the nurses (max 15/group) and motivated them to attend the education sessions on the wards.

Regarding the component ‘Goal setting with the patient’, nurses mentioned in the focus group interview goal setting as one of the key elements of the intervention when talking about FFCiH. The patient files showed that different short and long-term goals were set. Also, mobility, ADLs, and the evaluation of short-term goals were described in nurses’ reports. In addition, a few nurses said in the focus group interview that they set goals with the patient and some nurses let the patient set their own goals. Nevertheless, few nurses reported the goals in the nursing reports and if they did, it was not reported at the prescribed location in the patients’ file. Also, nurses said they set goals and used them in their care, but during observation, they showed they didn't use it as intended and the goals didn't guide their care. The multidisciplinary team was not involved in the goal-setting. The family was consulted only when a patient had cognitive problems. In general, nurses stated that they tend to set standard goals, they don't set goals together with the patient or family and nurses said they think for patients instead of with patients.

Regarding the component ‘Ongoing motivation and mentoring’ the logbook showed that nurses gave compliments, stimulated patients, explained why walking is important, and gave instructions about proper mobilization. Next to that, FFCiH coaches observed that nurses did walk more often with patients and were motivated to activate passive patients. FFCiH coaches were enthusiastic about FFCiH, made a plan to assure FFCiH, reminded and positively approached the team, and stimulated colleague nurses even when they resisted FFCiH.

Regarding reach, 54 Nurses (96.4%) on both wards attended at least 1 education session or participated in bedside teaching. A total of 40 (71.4%) nurses and nursing students followed education sessions, but the target for attendance set by the ward managers was not reached. After three months, 27 (48.2%) nurses and nursing students participated in bedside teaching (Table 4). At a cross-sectionally conducted patient file screening after three months, a goal was reported in 9 of the 16 (56.2%) electronic patient files.

### 3.2 Mechanism of impact

#### 3.2.1 Perceived facilitators and barriers regarding the components

Regarding the component 'Environmental and policy assessment’, the most important facilitator to apply FFCiH on the ward from the perspective of the environmental and policy assessment was the nurses’ daily confrontation with inactive patients. Another important facilitator was that nurses said during the focus group interview that they experienced FFCiH as an approach that fits with the principles underpinning their current working philosophy. In addition, one nurse said that other patient groups receiving care on their ward could also benefit from the FFCiH approach, indicating that nurses spontaneously generalized the application of FFCiH to other patient populations.

*“I applied it also on patients without a CVA. That is possible, right?” (Nurse in focus group interview)*

The FFCiH coaches suggested during a monthly meeting additional facilitators for implementation of FFCiH, like involving the FFCiH coaches in time and earlier in the process of implementation and giving them enough time to perform their role as FFCiH coaches. In addition, one barrier was identified. Nurses thought that the FFCiH approach was not suitable for the entire intended population, for example not for critically ill patients or patients who are about to go home.

Within the component ‘Education’ some perceived facilitators were found regarding the education sessions: the educational aspects were found interesting and educational, and the nursing team acknowledged the usefulness of FFCiH. On the other hand, barriers were that the nurses retrospectively experienced the education sessions as not always clear and the knowledge provided during the education sessions tended to fade over time. Furthermore, the nurses suggested during the focus group interview that they would like to be informed earlier in the process of implementation and educate the whole team. In one focus group interview, nurses suggested recurrent education sessions to optimize the sustainability of FFCiH over time. The FFCiH approach was not found to be new, nurses stated this both as facilitator and barrier. They stated that FFCIH was an addition to existing methods (facilitator), but that they already set goals and involved patients, and not always experienced FFCiH as useful (barrier).

*“We already do this, so we will continue to do this, but we now give it a different name.” (Nurse in focus group interview)*

Nurses showed contradicting opinions about the FFCiH guideline. Some considered the guideline as complete and no information could be skipped. But the same nurses also found the guideline not easy to use because of the large amount of information. A summary of the FFCiH guideline could be helpful according to the nurses. Regarding family participation, nurses suggested during the focus group interview some potential facilitators: timely informing the first contact person about FFCiH and providing an information folder to the family at patient admission.

Overall, in the component ‘Goal setting with the patient’, nurses and FFCiH coaches said during the focus group interview that they could have done better in setting goals. They stated that FFCiH is not in their routine and therefore was forgotten, care activities were taken over from patients without realizing it and FFCiH coaches did not remind their colleagues about FFCiH. An often mentioned barrier during the focus group interview for reporting the goals was the lack of perceived added value in the case of independent patients. Furthermore, reporting has a low priority and is perceived as energy-consuming because of the constant checklists that need to be administered. And even if nurses do see the added value to (report) goal setting, they lack integration in their daily routine. Facilitators for setting goals were the added value to the nursing care and setting the long-term goals, the focus that goal setting gives, and the fit to the senior-friendly certification of the participating hospital. Also, setting goals with the patient rather than for the patient was found to be attractive to the FFCiH coaches.

*“In particular, setting goals with the patient rather than for the patient is appealing to the working group” (Monthly meeting with coaches)*

Nurses suggested in the focus group interview that the whole team should start at the same time to stimulate goal setting. Nurses also suggested that a long-term goal could be set at the intake of admission to increase the involvement of the family. One nurse said that she likes to involve the family, but she felt family members doubt their own added value in the care of their loved one compared to the nurses who are the experts. Nurses also said that they already took into account patients’ capabilities and identified preferences from the admission notes. Moreover, information about the importance of activation during hospitalization appeared to be new for the family. To increase family involvement nurses suggested planning a meeting with a family member to evaluate the hospitalization and inform the family about the added value of FFCiH in a flyer.

Regarding the component ‘Ongoing motivation and mentoring’, during the focus group interview, the nurses said that reminders to set and report goals in the patients’ file and evaluation during implementation could better sustain the application of FFCiH. The lack of support for FFCiH from the team was often mentioned as a barrier, nurses said that the support of the team is necessary to apply FFCiH by themselves, the influence of the team is large both in positive and negative ways.

*“The prevailing culture applies when a lot of people do it, that‘s stimulating, you can't stay behind. And otherwise, when nobody does it, you think: well then I won't do it either.”(Nurse in a focus group interview)*

Furthermore, nurses noted during the focus group interview that they already involved patients in and stimulated ADLs and applied motivating interventions. Nurses had a few suggestions that might facilitate the ongoing motivation and mentoring: interim reminders and education to refresh the knowledge and skills of the nurses,

Also, nurses suggested a reminder in the patients’ file and that coaches should take over the role of the implementer. The researcher helped some nurses in setting goals with the patients. The nurses experienced the researcher as involved and found the bedside teaching helpful and the right example of how to apply FFCiH. The researcher took responsibility regarding FFCiH and had a clear role regarding FFCiH, but as a result, FFCiH remained a project of the researcher and not of the ward according to some of the FFCiH coaches. The FFCiH coaches said that they didn't know how to involve patients and their colleagues more and how to secure FFCiH in the ward.

### 3.3 Context

Factors that may affect the implementation are the fact that the family didn't have one point of contact, visited during busy hours for the nurses, and are also busy in their personal life. Things such as an increase in the number of beds on the wards, a recent move, and a high patient-nurse ratio for nursing students were aspects external to FFCiH that influenced the implementation process negatively.

### 3.4 Additions to the intervention

Based on these outcomes, we added some activities to the components to improve the FFCiH, no activities were removed (Table 1). The introduction of FFCiH should start with the component ‘Environmental and policy assessment’ by discussing the ward's policy and environment regarding supporting the physical activity of patients.

In the preparation of the education sessions, the ward manager, FFCiH coaches, and researcher should discuss when and how to inform the (multidisciplinary) team. Furthermore, it is important to emphasize the involvement of the ward manager, because of the perceived lack of support from the ward manager by nurses and FFCiH coaches during this study. We also suggest selecting coaches who are registered nurses with experience in care. Because of the challenging family participation by nurses, ‘emphasizing and addressing patient and family involvement’ is added to the education sessions in the component ‘Education’.

In the component ‘Ongoing motivation and mentoring’ should be defined how much time is expect to be needed for bedside teaching and what the best time is to provide it and by whom. Furthermore, not only the researcher but also FFCiH coaches should provide bedside teaching. Nurses need support in involving patients and families in their daily practice, for instance during bedside teaching. Furthermore, patient and family involvement should be embedded into the multidisciplinary collaboration on the ward. To increase patient and family involvement, written information about FFCiH and the advantages of physical activity during hospitalization should be added to the face-to-face information for patients and their families. To facilitate all these aspects, the ward manager needs to provide (time) conditions and support to the nursing team and coaches. Moreover, the ward manager needs to attend the monthly meetings with the FFCiH coaches and researchers.

## 4 Discussion

This study aimed to determine the feasibility of FFCiH on two wards in a general hospital. The implementation process went as planned, resulting in delivery of all components though not always as intended. Regarding the facilitators on the mechanism of impact, nurses welcomed the introduction of FFCiH since its fits with their focus on the recovery of the patient. Also, they acknowledge the importance of FFCiH and stated to know how to apply FFCiH. One of the perceived barriers to the mechanism of impact was that nurses lacked routine in applying FFCiH and prioritization to engage patients and families. Also, nurses experienced a lack of time and support for goal setting in daily care. Furthermore, some contextual factors, such as a high patient-nurse ratio, may have influenced the feasibility of FFCiH. These outcomes led to additions to FFCiH, to improve the intervention.

Several issues influenced the current feasibility of FFCiH. These issues need to be taken into account when implementing FFCiH on a larger scale. This study shows that nurses don't involve patients in goal setting and in half of the cases goals are not set at all. With this behavior, a key element of FFCiH is ignored, since goals need to be set in close collaboration with patients or families. Nurses might have felt no need to change anything in their behavior because some said they were already trained to engage patients in their ADLs as much as possible. Not involving patients is a missed opportunity, because literature shows that patient participation gives the patient control of their rehabilitation process ((Kitson et al., 2013b)), has an impact on enhancing adherence to treatment and advice (Aboumatarand Pronovost, 2012; Dulmen, 2011), and can shorten hospital stay (Ekman et al., 2011). Next to that, nurses fell back into old routines regarding motivating patients for goal setting and physical activity, as soon as they were not reminded anymore. The relapse might be explained by the way bedside teaching was performed, which was insufficiently tailored to the size of the team and their needs. Literature shows that relapse in performance is often a reoccurring aspect in an implementation process. To prevent relapse different strategies can be used such as reminders, ongoing consultation, clinical supervision, and facilitation (Powell et al., 2015). This relapse was also found in a pilot study of FFC on an acute care trauma unit (Burket et al., 2013). This underpins the importance to allocate enough time for bedside teaching and thorough consideration about when to do this and by whom.

Additionally, a discrepancy was observed between what some nurses stated is best to do and what they were doing. Nurses said that they involve patients and focus on stimulating mobilization and the independence of the patients, but the observations showed they did not always involve the patient in goal setting and that they take over actions in busy shifts. A similar gap between saying and doing was observed in a surgical hospital setting that showed that although the nurses had the theoretical knowledge about pain management, they did not always use this knowledge during daily practice (Dihle et al., 2006). The authors suggested this might be because reflecting upon their own experience and theoretical knowledge had not become part of their routine (Dihle et al., 2006). This could also be a plausible explanation for our study since reflection upon own behavior was also seldom seen. The majority of the nurses predominantly identified barriers external to themselves for the implementation of FFCiH. For example, bedside teaching is one of the possibilities for nurses to reflect upon their behavior, but some nurses stated that because of the bedside teaching the researcher remained the owner of FFCiH. This resulted in less support to FFCiH by the nurses themselves.

One of the most mentioned barriers external to the nurses themselves was the lack of time, which according to the nurses leads to less time for applying goal setting, physical activity for patient and family participation. In an observational study where nurses stated that allowing the patient to get dressed by themselves requires time which they do not have in a busy shift (Loft et al., 2017). Furthermore, in a cross-sectional survey, the nurses felt they had to prioritize their daily nursing care activities due to lack of time (Ball et al., 2014). Prioritizing care activities was also done by the nurses in the current study giving medical-related tasks priority. This is supported by two systematic reviews on missed nursing care tasks in the hospital, which revealed that documentation of nursing care, mobilization, and education were some of the least performed activities (Jones et al., 2015; Mandal et al., 2020). Another possible explanation for the time-related barrier might be that the nurses still not perceived the benefits of FFCiH outweighed the disadvantages of the extra time it takes to encourage the patient. This might emphasize the need for extra support and convincing information (Powell et al., 2012).

Our study also showed that family participation was challenging. Nurses attributed this among other things to lack of time during the busy evening shifts. However, lack of priority may also be an explanation, because of the many reasons mentioned why nurses did not involve family. Other researchers studying family participation confirm the challenge of family participation during hospital stays (Ball, 2014; Mackie et al., 2018). Nurses’ actions appeared to be influenced by what they considered to be the priorities of the unit and hospital (Ball, 2014; Mackie et al., 2018). They also found that nurses only want to engage with family if it saves them time and if not, they restrict family participation (Mackie et al., 2018). This might have been the case in our study as well. Family's resistance against physical activity was revealed to be a barrier in our study as well. According to the nurses, this was due to the family's opinion or lack of knowledge about the benefits of physical activity. However, taking into account the lack of time nurses experienced to involve family, it might be questioned to what extent the family was adequately informed about the benefits of physical activity by the nurses.

Our findings also show that the FFCiH coaches didn't know how to secure FFCiH and not all FFCiH coaches were able to fulfill their role on the wards. Next to that, nurses experienced little support from the ward manager and they experienced no consequences when not applying FFCiH. It is thus questionable whether the ward managers were sufficiently aware of the importance of their role in the intervention. Literature shows that an important characteristic for influencers, such as the FFCiH coaches and ward manager, is to have experience as a nurse, besides other skills as the ability to give feedback, availability of time, and a positive attitude (Huybrecht et al., 2011). This is confirmed in two hand hygiene studies with nurses, in which a team and leaders-directed strategy, containing social influence and leadership, was positively correlated to hand hygiene compliance (Huis et al., 2013a, 2013b). The leadership strategy consisted of ward managers’ discussions of compliance with team members, holding nurses accountable for their behavior, and designating hand hygiene as a high ward priority (Huis et al., 2013a, 2013b). This kind of involvement and commitment of formal leaders was not seen in our study, which might explain the low compliance to FFCiH in the nurses’ daily care. Although we incorporated these aspects in our strategies, the sub-optimal role of the FFCiH coaches with regards to social influence and leadership can relate to the relapse seen in our study. This, even more, emphasizes the importance of selecting coaches who are nurses with experience in care and who are informal leaders in the team.

Nurses also mentioned the lack of involvement of the multidisciplinary team in FFCiH as a barrier. Some of the multidisciplinary team members attended the education sessions, but nurses did not see any further collaboration. A multidisciplinary approach for hospitalized patients is a basic ingredient to prevent functional decline (Hoogerduijn et al., 2014). Next to that, when the compliance of nurses to FFCiH is higher, they could also involve the multidisciplinary team better in the patients’ goals.

### 4.1 Strengths & limitations

The use of a well-known framework for process evaluation, the MRC-framework, and different data collection methods is a strength of this study. The combination of quantitative and qualitative methods resulted in a detailed understanding of the feasibility of FFCiH (Cheryl and Creswell, 2016; Moore et al., 2015). With our findings regarding the description of the intervention, implementation process, and mechanism of impact we provided a thorough insight into the feasibility of FFCiH.

Also, some limitations should be noted. First, self-reported data were used in this study which can lead to socially desirable answers from the nurses regarding the activities performed (Antinaho et al., 2017) or overestimating their performance (Ehrlinger et al., 2008). This also applies to the observations, in which nurses might have changed their behavior while being observed or might have avoided being observed (Antinaho et al., 2017). However, we strongly improved our results by combining the qualitative methods with quantitative methods to check some of the findings we observed. For example, nurses said they set goals and used them in their care, but during observation, they showed they didn't use it as intended and the goals weren't a guide for their care.

Second, a limitation was the dual role of the researcher combining a key role in the introduction of FFCiH with the role of data collector. The best example of this dual role was bedside teaching. This was one of the activities as part of the implementation element of the component ‘Education’, but it was also used as an opportunity to observe the nurses' behavior during care activities. The researcher, therefore, was able to immerse herself in the daily practice of the wards and get a full understanding of the performance of the nurses and perceived barriers, which is a strength. However, this also may have resulted in biased results since it might result in an increased subjectivity of the investigator (Cheryl and Creswell, 2016). We, therefore, made the following efforts to ensure objectivity. Member checking was used to determine the trustworthiness of the focus group interviews. Furthermore, the analyses of the qualitative data were performed by two researchers separately, a third researcher was consulted to reach consensus, and findings were established based on converging several sources of data. These efforts might be considered as adding to the validity of the study (Cheryl and Creswell, 2016).

### 4.2 Implications for practice and further research

The findings of our study can provide recommendations for future implementation of FFCiH in the Dutch hospital setting. Especially patient and family involvement within all components of FFCiH needs to be emphasized when applying FFCiH. Also, (time-related) barriers and the underlying explanations need more attention and need to be discussed regularly, for instance during the follow-up education sessions. These sessions should be planned depending on the possibilities of the ward to prevent nurses have ‘no time’. Ward managers and coaches need to be more actively involved in the implementation process.

Feasibility studies are needed to evaluate whether the adaptation of an intervention, such as FFCiH, to a specific setting was successful. Our study method can be used to assess the feasibility of FFCiH in other settings or countries. We showed that a lot of elements are feasible, but need to be embedded at the ward level. This is also described in the intervention itself.

As a next step in the development of complex interventions of the MRC-framework (Craig et al., 2008), FFCiH should be evaluated on a larger scale. The effectiveness of FFCiH should be assessed before implementation studies are conducted. Next to that, the change process of both the patients’ and nurse's perspectives should be studied to provide valuable insight into why an intervention fails or has unexpected consequences (Craig et al., 2008). Lastly, the cost-effectiveness can be assessed.

## 5 Conclusion

In this study, we revealed that overall FFCiH is feasible for the Dutch hospital setting. Furthermore, our findings place a strong emphasis on team involvement, nursing leadership during the implementation, and the involvement of patients and their family to optimize future implementation of FFCiH in Dutch hospitals.

## Funding sources

The author(s) disclosed receipt of the following financial support for the research, authorship, and/or publication of this article: This work was supported by The Netherlands Organization for Health Research and Development (ZonMw) (Grant # 80–80705–98–025).

## Data availability

Data is available upon request by the corresponding author.

## Declaration of Competing Interest

None
